# Supplementary material for: Tead1a Initiates Transcriptional Priming Through the TEAD1a/YAP‐Notch1‐Spi1/Cebpα Axis to Promote Neutrophil Fate
Source: Adv Sci (Weinh). 2025 Aug 28;12(41):e05441. doi: 10.1002/advs.202505441 (PMC12591127; doi:10.1002/advs.202505441)
Supplement: Supplementary file 4 — Supplementary Dataset [file ADVS-12-e05441-s004.zip › 3-3.zip!/3-3/02-3.cluster/cluster7/Enrich/ego_CC.pdf]

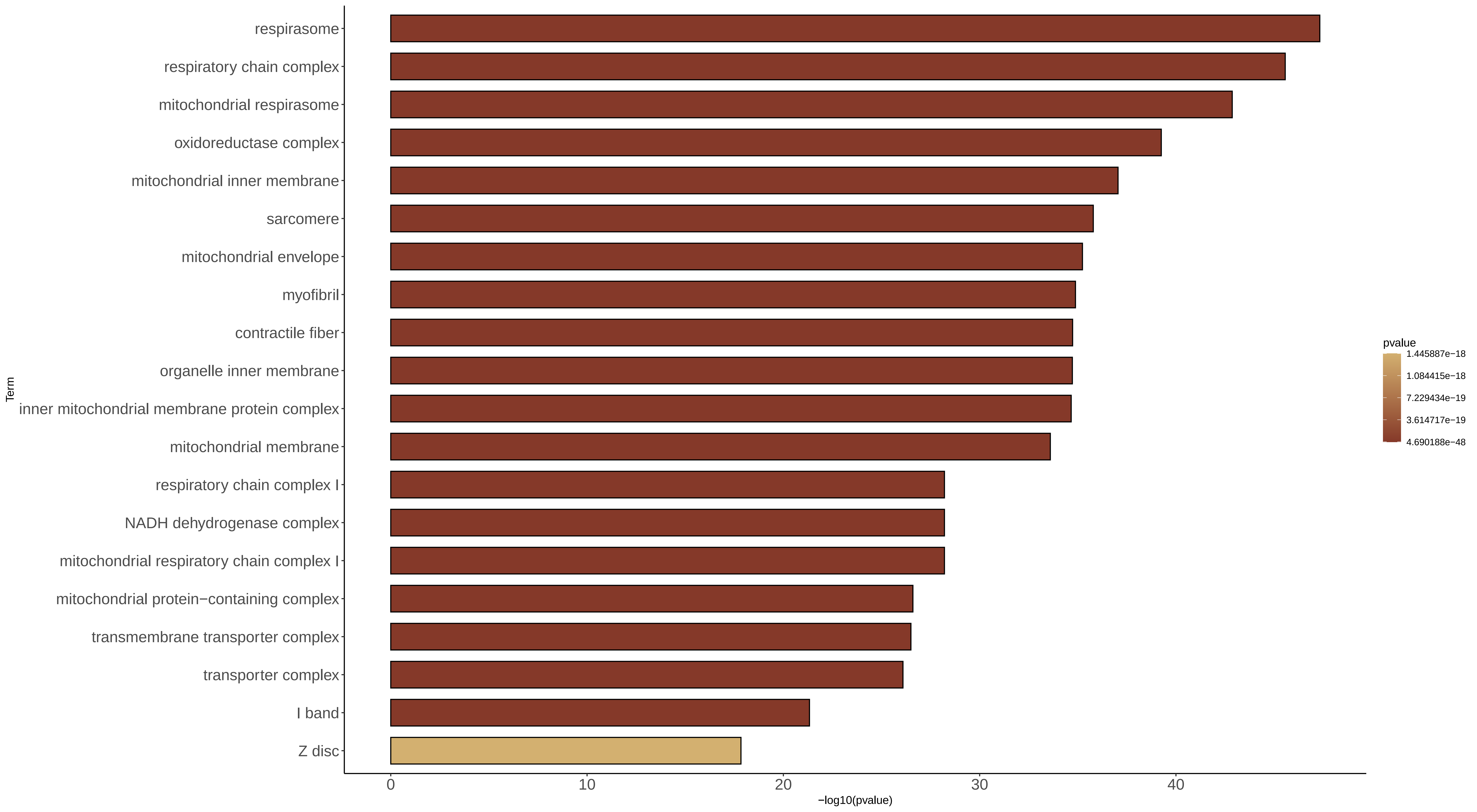
respirasome
respiratory chain complex
mitochondrial respirasome
oxidoreductase complex
mitochondrial inner membrane
sarcomere
mitochondrial envelope
myofibril
contractile fiber
organelle inner membrane
inner mitochondrial membrane protein complex
mitochondrial membrane
respiratory chain complex I
NADH dehydrogenase complex
mitochondrial respiratory chain complex I
mitochondrial protein−containing complex
transmembrane transporter complex
transporter complex
I band
Z disc
Term
0
10
20
30
40
−log10(pvalue)
pvalue
1.445887e−18
1.084415e−18
7.229434e−19
3.614717e−19
4.690188e−48
